# Polyanion order controls liquid-to-solid phase transition in peptide/nucleic acid co-assembly

**DOI:** 10.3389/fmolb.2022.991728

**Published:** 2022-11-14

**Authors:** Christella Gordon-Kim, Allisandra Rha, George A. Poppitz, Jillian Smith-Carpenter, Regina Luu, Alexis B. Roberson, Russell Conklin, Alexis Blake, David G. Lynn

**Affiliations:** ^1^ Department of Chemistry, Emory University, Atlanta, GA, United States; ^2^ Children’s Health of Orange County, Research Institute, Orange, CA, United States; ^3^ Department of Biology, Emory University, Atlanta, GA, United States

**Keywords:** systems chemistry, templated co-assembly, amyloid plasticity, systems analyses of co-assembly, nucleation and propagation of paracrystalline assemblies, templating cross-*β* peptide architectures with nucleic acids

## Abstract

The Central Dogma highlights the mutualistic functions of protein and nucleic acid biopolymers, and this synergy appears prominently in the membraneless organelles widely distributed throughout prokaryotic and eukaryotic organisms alike. Ribonucleoprotein granules (RNPs), which are complex coacervates of RNA with proteins, are a prime example of these membranelles organelles and underly multiple essential cellular functions. Inspired by the highly dynamic character of these organelles and the recent studies that ATP both inhibits and templates phase separation of the fused in sarcoma (FUS) protein implicated in several neurodegenerative diseases, we explored the RNA templated ordering of a single motif of the Aβ peptide of Alzheimer’s disease. We now know that this strong cross-*β* propensity motif alone assembles through a liquid-like coacervate phase that can be externally templated to form distinct supramolecular assemblies. Now we provide evidence that structured phosphates, ranging from complex structures like double stranded and quadraplex DNA to simple trimetaphosphate, differentially impact the liquid to solid phase transition necessary for paracrystalline assembly. The results from this simple model illustrate the potential of ordered environmental templates in the transition to potentially irreversible pathogenic assemblies and provides insight into the ordering dynamics necessary for creating functional synthetic polymer co-assemblies.

## Introduction

Cellular RNA is generally sequestered early within liquid-like protein coacervates named ribonucleoprotein granules (RNPs) (Banani et al., 2017; Tauber et al., 2020; Fomicheva and Ross, 2021). The functions of these cellular membraneless organelles range from storage granules to processing hubs chaperoning mRNA through the critical stages of cellular stress, division, and differentiation (Alberti et al., 2017; Shin and Brangwynne, 2017; Alberti et al., 2019; Tauber et al., 2020). The multifaceted functions that RNP granules play depends on dynamic structural plasticity of these co-assemblies (Banani et al., 2017; Boeynaems et al., 2019; Conicella et al., 2020; Dutagaci et al., 2021; Gordon et al., 2021; Hallegger et al., 2021; Carey and Guo, 2022). For example, the cooperative interactions between arginine residues of the RNA binding domain and the tyrosine residues from the prion-like domains (PLD) of FUS are critical for the dual effects of ATP concentrations that both induces and inhibits initial phase transitions at different concentrations by controlling these interactions (Wang et al., 2018; Ren et al., 2022).

**FIGURE 1:**
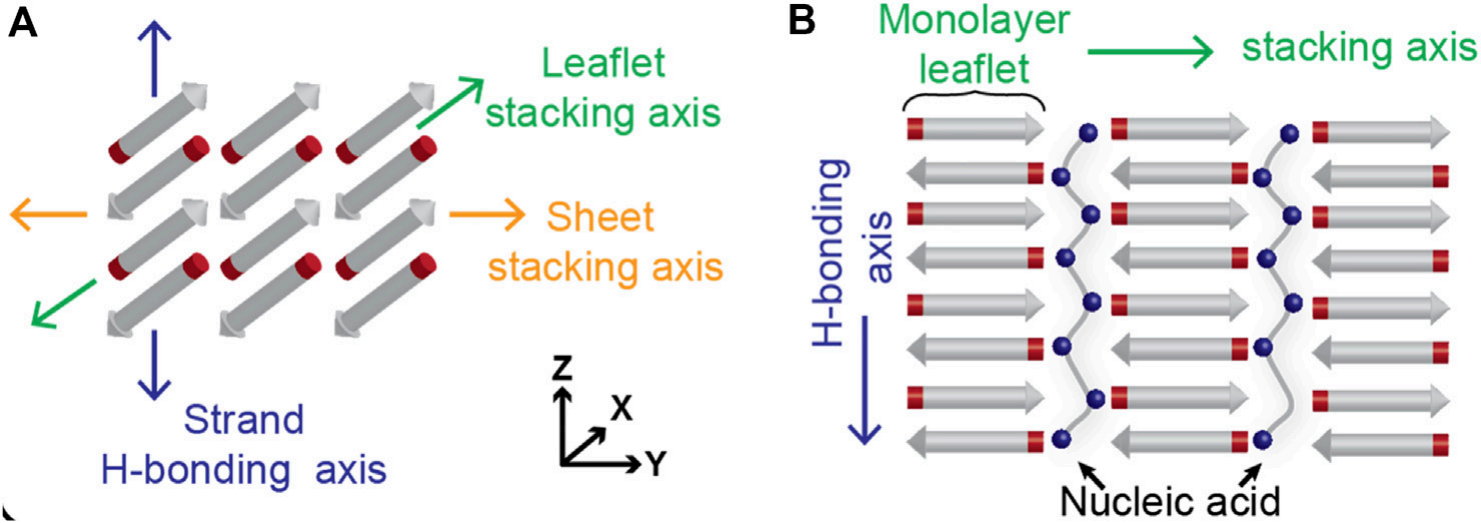
Cross-*β* architecture allows for packing of other polymers in between the leaflets. Hydrogen bonding across amides from consecutive peptide strands contribute to leaflet formation in the *z*-axis, while side chain interactions contribute to the stacking of *β*-Sheets along the *y*-axis **(A)**. The leaflets, where the peptide termini are positioned, have been shown to accommodate polyanionic species like polyphosphates and oligonucleotides **(B)**.

The nucleating core of the Alzheimer’s disease-associated Aβ42 peptide, the sequence KLVFFAE, has been extensively studied as a separate peptide and shown to have strong cross-*β* propensity: independently undergoing two-step nucleation that includes condensation and subsequent self-assembly into cross-*β* fibers, which can allow for the inclusion of other polymers between the leaflets (Figure 1). There is now evidence that these peptide condensates are preordered within the condensates for nucleation, highlighting the remarkable potential environmental control over the final assembled morphology (Mehta et al., 2008; Childers et al., 2012; Mehta et al., 2013; Uversky, 2013; Liang et al., 2014; Arai et al., 2015; Mollica et al., 2016; Hsieh et al., 2017a; Kim and Han, 2018; Gordon et al., 2021; Hallegger et al., 2021). Indeed, subtle changes in amino acid sequence greatly impact the rates of nucleation and propagation along the three potential cross-*β* assembly growth planes. The Aβ motif congener Ac-KLVIIAG-NH_2_ (Pep-KG) is derived from KLVFFAE, with the phenylalanine dyad replaced by isoleucine and the terminal glutamate replaced by glycine. Pep-KG was designed to have a lower propensity for nucleation relative to KLVFFAE by replacing the phenylalanine with isoleucine residues and only retaining a single cationic residue for charge-driven phase separation templated by polyanions. These changes allowed for nucleic acid passivation of the leaflet interface by nucleic acids with the subsequent assembly of distinct antiparallel *β*-Sheets organized as multilamellar cross-*β* nanotubes (Rha et al., 2020). RNA, DNA, and polyphosphate (p50) all nucleate assembly, consistent with electrostatics playing significant roles in these two-step transitions much like ATP with FUS (Hsieh et al., 2017b). While the final peptide cross-*β* assembly with phosphate pacification are well ordered, the nucleic acid backbone is ordered electrostatically in respect to the peptide assembly, with consistent distances between Pep-KG lysines and nucleic acid phosphates as shown by previous solid state NMR data (Rha et al., 2020).

Given the role of ATP as a trivalent binder regulating FUS assembly and the range of cellular processes that depend on RNA structural dynamics within RNP granules, we have now explored this single motif as a reporter for the role that nucleic acid conformations may play in the two-step nucleation process (Brazda et al., 2011; Flores and Ataide, 2018; Sanchez de Groot et al., 2019; Chilinski et al., 2022; Saurabh et al., 2022). Nucleic acid conformations dictate the ordering of phosphate charges along the backbone and significantly impacts the initial nucleation of supramolecular co-assembly. This ordered nucleation sets the stage for the propagation of unique paracrystalline order. Using this insight, we have designed nucleic acid-peptide chimeras to create novel architectures that exploit both the plasticity of the peptide motifs as well as the nucleic acid templates. These results provide a foundation for strategies focused on the construction of nucleic-acid-based functional materials with cross-*β* assembles and could further inform the diverse and dynamic controls underlying ribonucleoprotein granule function. This knowledge will prove useful in the rapidly evolving field of biomaterials, drawing parallels between exploiting the energetics of coacervate materials and bottom-up approaches for materials design.

## Materials and methods

### Synthesis of Ac-KLVIIAG-NH_2_ (Pep-KG) and Ac-ELVIIAG-NH_2_ (Pep-EG)

Pep-KG was synthesized on Rink amide-MBHA (Anaspec) solid support *via* a CEM Liberty Blue Automated Microwave Peptide Synthesizer (Serial # LB2447) with 1M N,N′-Diisopropyl carbodiimide (DIC; CAS# 693-13-6 AAPPTEC) as the activator, Oxyma Pure (CAS# 3849-21-6 CEM) as the activator base, and 20% Piperidine (CAS# 110-89-4 Sigma-Aldrich) as the deprotection solution. Amino acids were coupled using 0.25 mmol standard coupling (75°C 210 W for 15 s followed by 90°C 30 W for 110 s) and deprotected using 0.25 mmol standard deprotection (75°C 175 W 15 s, 90°C 30 W 50 s). The amino acids used were as follows: fmoc-boc-lysine, fmoc-leucine, fmoc-valine, fmoc-isoleucine, fmoc-alanine, fmoc-glycine, fmoc-boc-glutamic acid, and each one dissolved in dimethylformamide (DMF; CAS# 68-12-2 Sigma-Aldrich). The N-terminus of both Pep-KG and Pep-EG were acetylated with a 20% acetic anhydride (CAS# 108-24-7 Sigma-Aldrich) in DMF solution and the 0.25 mmol N-terminal acetylation method (60°C 50 W 30 s, 25°C 0 W 30 s, 60°C 50 W 30 s, 25°C 0 W 30 s). Upon completion of synthesis, the resin beads were rinsed with dichloromethane (DCM) then let dry *via* vacuum filtration for cleavage from solid support which was carried out using a cocktail of 9:0.5:0.3:0.2 ratios of trifluoroacetic acid (TFA CAS# 76-05-1 Chem Impex)/thioanisole (CAS# 100-68-5 Sigma-Aldrich)/1,2-ethanedithiol (CAS# 540-63-6 Sigma-Aldrich)/anisole (CAS# 100-66-3 Sigma-Aldrich), where 10 ml of the cocktail was used in each of two vials and the 0.25 mmol of peptide attached to resin was evenly distributed across the two vials. The resin beads were submerged in the cocktail and were continuously perturbed using an orbital shaker at low intensity for to allow for homogeneous coverage of the beads with the cleaving reagents, and the reaction vessels were left for 3 h at room temperature. Upon completion, the beads are filtered from the peptides *via* gravity filtration immediately into cold (−20°C) ether (CAS# 60-29-7 Fischer Scientific). At this point, the ether should become warm and cloudy due to mass precipitation of the peptides, and the mixture is then spun down at 4000 RCF for 15 min at 4°C to improve precipitation. The ether supernatant was discarded, and the gel-like pellet was washed with more cold ether for centrifugation, a process that is repeated twice more. The pellet was stored *in vacuo* pending HPLC purification and then a standard desalting protocol, as described in the Supplementary Material document.

### Preparation of GQ/Pep-KG conjugate

Desalted Pep-EG (20 mg) and 33.5 mg N-hydroxysuccinimide (CAS# 6066-82-6 Sigma-Aldrich) were dissolved in minimal amount of DMF (approximately 2 ml). 55.8 mg EDC (1-Ethyl-3-(3-dimethylaminopropyl)carbodiimide; CAS# 25952-53-8 Sigma-Aldrich) was added afterward, and the solution were left to stir at room temperature overnight, up to 36 h. Completion of this reaction generated an imide-peptide species at 851 Da (Supplementary Figure S1). 100 μl 0.2M NaHCO_3_ was then added to the solution, and 0.15 mmol propargylamine (CAS# 2450-71-1 Sigma-Aldrich) was added immediately after the pH was raised. This reaction was left to stir for 5 h and propargylamine coupling, which generates Ac-E_m_LVIIAG-NH_2_, was confirmed *via* mass spectrometry (Supplementary Figure S2). This crude product was used without further purification for conjugation with 5′-azide modified GQ DNA (T4GGTG4TGG) *via* azide-alkyne click reaction using 4 nmol GQ DNA, excess of Ac-E_m_LVIIAG-NH_2_ (4 μl), 4 μl 2M triethylamine-acetate buffer at pH 7, and 4 μl of saturated ascorbic acid for activation of 10 mM Cu(II)-TBTA in 55% aqueous DMSO (Lumiprobe, MD, United States) was added last (4 μl). This reaction was run at room temperature for up to 48 h and the peptide/DNA conjugate was precipitated with ethanol. Despite the excess of peptide, only peptide/DNA conjugates were recovered using this protocol involving 2 ml of 2M MgCl_2_ and a fourfold excess of 200 proof ethanol. The solution was shaken vigorously prior to placing in an ice bath for 1 hour. As the solution became turbid during the incubation period, the sample was spun down at 13,000 rpm for 20 min to give a gel-like pellet. After decanting the supernatant, the pellet was resuspended and washed with 70% ethanol and then spun down once more at 13,000 rpm for 10 min to give the peptide/DNA chimera. The ethanol was decanted, the pellet was analyzed by Urea-PAGE electrophoresis and mass spectroscopy, and the final sample was stored *in vacuo* until co-assembly with Pep-KG.

### Assembly of Pep-KG with and without templates

All assembled samples were prepared using desalted Pep-KG following HPLC purification. Pep-KG was assembled at a final concentration of 1 mM both in the presence and the absence of polyanionic templates. Templates were added to satisfy a 1:1 charge ratio with Pep-KG, which contributes a single positive charge. Assemblies were done with the following charge assumptions: the Drew-Dickerson dodecamer single stranded sequence d(CGCGAATTGCGC) contributed a charge of −12, whereas the double stranded counterpart of the same sequence had −24 charges. Both dA3 and trimetaphosphate contributed a −3 charge each. A solution of double the concentration of each template, relative to the template’s final concentration, was prepared using 40% MeCN, where an equal volume of 2 mM Pep-KG in 40% MeCN was then added to the template solutions that were pre-incubated at 37°C. As an example, an initial solution of 400 μl 0.66 mM trimetaphosphate was prepared in 40% MeCN, where 400 μl 2 mM Pep-KG in 40% MeCN was added to it. All samples were incubated at 37°C throughout the assembly times of up to 2 weeks.

### ThT fluorescence assay

Samples for Thioflavin T fluorescence analyses were prepared by combining 74 μl of each sample, 100 μl of 40% acetonitrile, and 1 μl of 10 mM Thioflavin T (CAS# 2390-54-7 purchased from Sigma-Aldrich) and measured in the wells of a 96 well plate (Microplate, 96 well, PS, F-bottom, μCLEAR, black, med. binding, Greiner Bio-one). Thioflavin T fluorescence was determined with a BioTek Synergy Mx plate reader (Serial# 250843) every 15 min for up to 24 h, with short shaking before each read. The excitation wavelength was 444 nm and fluorescence was measured at 484 nm. A well containing 1 μl 10 mM ThT in 40% MeCN and 174 μl 40% MeCN was used as a baseline. The plate was held at 37°C for all 24 h.

### Sample preparation for TEM imaging

Each selected sample (8 μl) was pipetted onto a carbon-film coated, 200 mesh copper grid (CF200-Cu purchased from Electron Microscopy Services) The sample was then negative stained with 8 μl of the supernatant of a 2% w/v Uranyl acetate in water solution that had been centrifuged at 12,000 RCF for 10 min (CAS# 541-09-3 purchased from Electron Microscopy Solutions). Loaded electron microscopy grids were visualized with a Hitachi HT7700 transmission electron microscope at 80 kV.

### Sample preparation for EFM imaging

Aliquots (10 μl) of GQPC assemblies in 40% acetonitrile were deposited on gold film upon Si/SiO_2_ substrate and then dried over 12 h. All micrographs were taken by Park System XE-100 AFM in tapping mode. To probe for charge surface of our assemblies, a charge bias of +1 V was applied between the electrically conductive Pt-Ir coated tip, with 2.7 N/m force constant, (Mountain View, CA, AppNano) and the sample.

### Characterization of assemblies *via* circular dichroism spectroscopy

Circular Dichroism (CD) analyses were recorded on a Jasco-810 Spectropolarimeter. Samples were micro-pipetted onto a 50 μl Hellma Analytics quartz cell with a 0.1 mm path length (Model # 106-0.10-40). Spectra were measured by averaging three scans from 260-190 nm with a 0.2 nm data pitch and 100 nm s^−1^ scanning speed. Molar ellipticity was calculated with the equation [θ] = θ/(10 × c × l) where c is the peptide concentration in moles/L and l is the pathlength of the cuvette (cm).

### Circular dichroism melting experiments

43 μl of each sample was loaded onto a 50 μl Hellma Analytics quartz cell with 0.1 mm path length (Model # 106-0.10-40) while taking care to minimize bubbles inside the cuvette, and the edge of the cuvette was wrapped in parafilm to minimize evaporation. Each melting trial was conducted using a Jasco J-1500 (Serial #B043361638) Spectropolarimeter starting at 37°C and ending at 92°, changing 1°C every minute and accumulating three scans from 260 to 190 nm every 5°C interval. The data pitch was 0.2 nm, the scanning speed used was 200 nm min^−1^.

### Fluorescence spectroscopy for analyzing ISCH-oa1 binding

ISCH-oa1 were all measured at 1 μM final concentration, both in the presence and in the absence of peptides or GQ DNA. Each sample was measured in 3 mm path length quartz cuvette (Müllheim, Germany, Hellma Analytics) using Cary Eclipse Fluorescence Spectrophotometer (Santa Clara, CA, Agilent). Excitation was set to 550 nm, and emission spectra were acquired from 560 nm to 700 nm. Raw data were normalized to f/f_0_, where f is fluorescence of each sample, and f_0_ is fluorescence of ISCH-oa1 probe at 1 μM concentration.

## Results and discussion

### dsDNA efficiently templates two-step nucleation for cross-*β* peptide assembly

**FIGURE 2:**
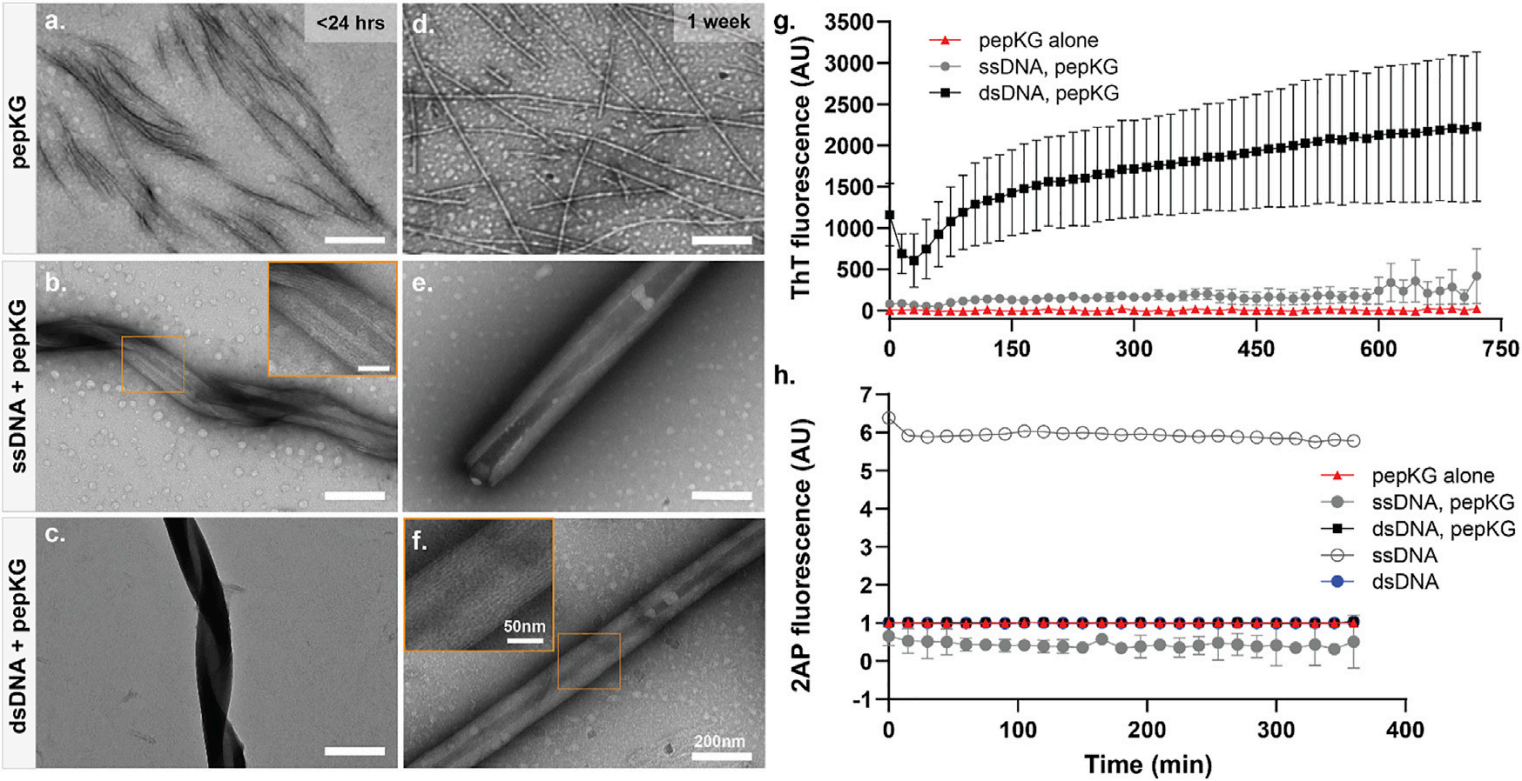
Pep-KG assembly rate is accelerated when templated with dsDNA. TEM image panels **(A–C)**: Pep-KG assembled alone, Pep-KG assembled with ssDNA, and Pep-KG assembled with dsDNA, respectively, within 1 day of initial dissolution. Panels **(D–F)** were taken after 1 week of incubation. All peptides and DNA templates were dissolved in 40% MeCN at 1 mM Pep-KG concentration and continuously incubated at 37°C. Measurements of ThT fluorescence **(G)** indicates cross-*β* growth of the assembly within the first 12 h and **(H)** 2AP fluorescence within the first 6 h of assembly. Each fluorescence measurement was done at 37°C incubation across all timepoints. All error bars are 95% CI values and all samples for 2AP and ThT fluorescence are *n* = 3.

It has been demonstrated that charged monomer sequence patterns can influence complex coacervate formation in synthetic polymers, but how nucleic acid polymer conformations impact peptide coacervation remains unknown. To investigate the role higher-order nucleic acid structure plays in nucleating 2-step peptide assembly, the single stranded dodecamer (5′-CGCGAATTCGCG-3′, ssDNA) and its Drew-Dickerson B-DNA duplex (dsDNA) were each incubated with the single sticker/spacer motif, Pep-KG (Ac-KLVIIAG-NH_2_) (Dickerson and Drew, 1981a; Dickerson and Drew, 1981b; Drew and Dickerson, 1981; Drew et al., 1981; Marky et al., 1983). The structures formed by this DNA sequence has been extensively studied for decades and is short enough to limit conformational sampling throughout the studies. Furthermore, previous studies have demonstrated that the nanotube morphology are independent of oligonucleotide sequence and length, provided that there are at least six consecutive nucleotides (Rha et al., 2020). As shown in Figure 2A top panel, Pep-KG alone undergoes 2-step nucleation, transitioning through initial condensates to fibrous assemblies within a day of dissolution and remains as such (Figure 2D) indefinitely, as previously reported (Rha et al., 2020). In comparison, when Pep-KG is assembled in the presence of DNA templates at a 1:1 charge ratio, the rate of assembly is dramatically accelerated as reported by thioflavin T (ThT) fluorescence (Figure 2G). While both dsDNA and ssDNA templates dramatically impact Pep-KG assembly, dsDNA rapidly templates helical ribbons while ssDNA shows much greater heterogeneity at early time points (Figures 2B,C). However, with longer incubation times, both ssDNA and dsDNA template the assembly of similar multilamellar ribbon morphology that ultimately transitions into nanotubes (Figures 2E,F).

To evaluate whether the initial coacervation step might be impacted by the charge ordering of the template, the fluorescent probe 2-aminopurine (2AP) (Ex. 310 nm/Em. 370 nm) was synthetically incorporated into the center of the dodecamer sequence, 5′-CGCG(2AP)ATTCGCG-3’. As shown in Figure 2H, the fluorescence is immediately quenched in all the peptide co-assemblies to a level similar to that of the dsDNA, significantly less than the ssDNA alone. These data establish that ssDNA does not self-assemble under these conditions, and rapidly condenses with the oppositely charged peptide. This immediate 2AP quenching in ssDNA is consistent with long, synthetic, same-charge monomers in polymers exhibiting strong charge interactions in complex coacervate condensation (Chang et al., 2017).

**FIGURE 3:**
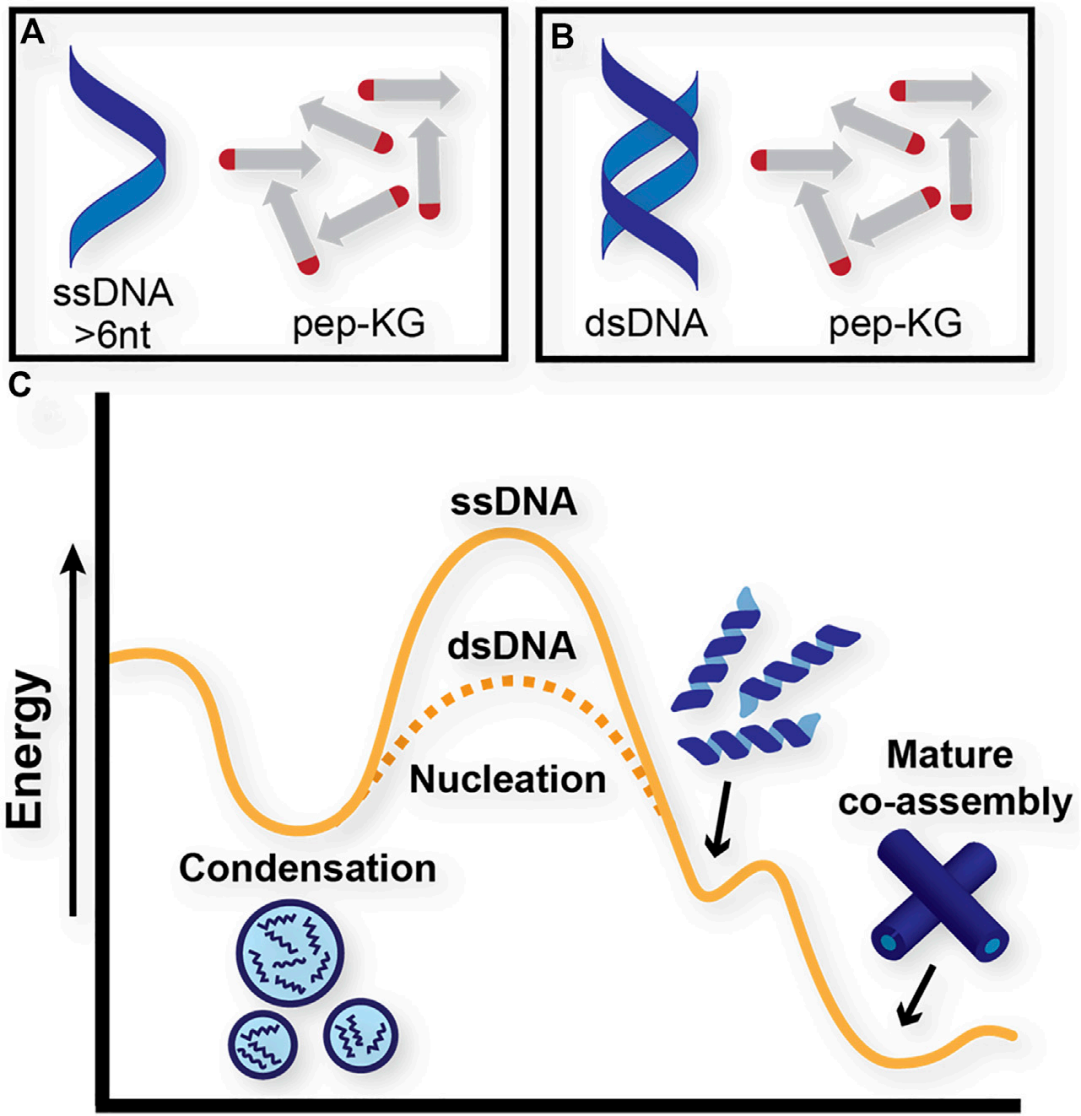
Proposed energy diagram of distinct pathways that Pep-KG may undergo through context-dependent co-assemblies. **(A)** and **(B)** denote Pep-KG assembly templated by ssDNA and dsDNA, respectively. Proposed energy diagram **(C)** illustrates dsDNA templates lower the energetics of nanotube assembly relative to ssDNA templates, as demonstrated by ThT fluorescence experiments.

The dsDNA template rapidly nucleates and propagates paracrystalline peptide assembly (Figure 2G), but the initial ThT fluorescence drops during the first hour, and then grows cooperatively. This initial drop could be the result of breathing modes and fraying at the ends of the template as it enters the peptide condensate (Leroy et al., 1988; Peyrard et al., 2009). The apparent autocatalytic growth has been previously seen with rearrangements that alter ThT binding as the growing assembly moves beyond the initial biomolecular condensate (Hsieh et al., 2017a; Rengifo et al., 2020). The ssDNA also templates peptide ordering, but only beginning after 6 h of incubation as monitored by ThT fluorescence (Figure 2G). Taken together, these results indicate that although both single-stranded and double-stranded DNA function as polyanionic templates capable of nucleating peptide assembly, double-stranded DNA accelerates the formation of paracrystalline assemblies. Given what we know about the assembly pathway of Pep-KG, we believe this to be because dsDNA is more effectively recruiting Pep-KG monomers within the particle phase and more rapidly forming a stable nucleus which then propagates out of the particle into ribbons and finally nanotubes (Figure 3).

### Electrostatic ordering underlies template effectiveness

**FIGURE 4:**
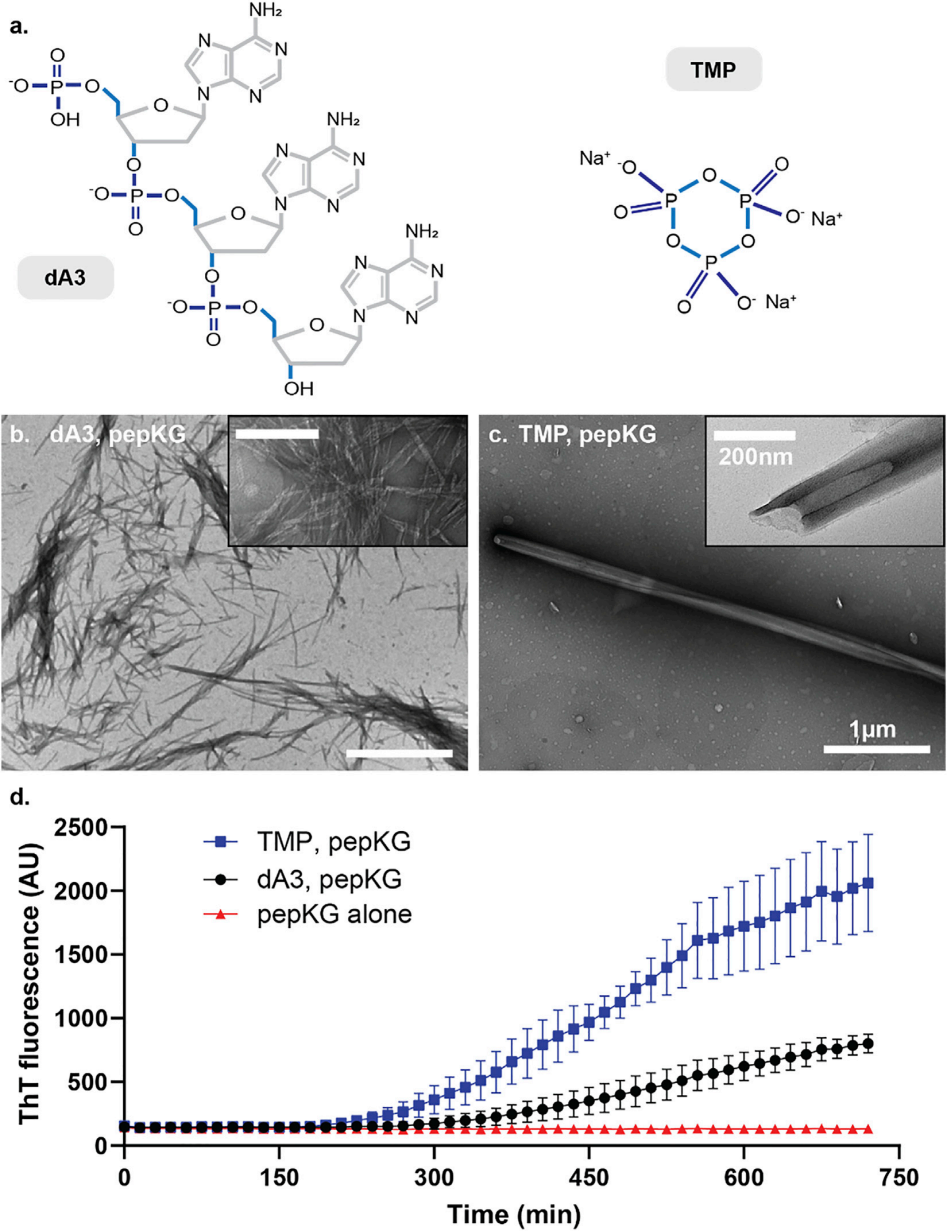
**(A)** Structures of A3 (left) and TMP (right). Micrographs of Pep-KG assembled in the presence of A3 **(B)** and in the presence of TMP **(C)**. Both images were taken within 1 day of dissolution in 40% MeCN at 37°C. Both samples consisted of 1 mM Pep-KG and 333 μM dsDNA and 333 μM TMP. **(D)** ThT fluorescence of Pep-KG/dA3 and Pep-KG/TMP samples within the first 12 h. Error bars represent 95% CI values, and all samples are *n* = 3.

The dsDNA template contains twice the number of phosphates as the ssDNA template, and this difference may contribute to the difference in nucleation efficiency. However, the number of charges is not the only difference when comparing dsDNA to ssDNA, as the duplexed DNA also exhibits a significant surface area increase, which impacts the spatial ordering of the charges. Thus, to determine whether the number of phosphates or their order contributes most significantly to nucleation efficiency, we compared dA3, the 5′-phosphorylated linear adenine ssDNA, and trimetaphosphate (TMP), a constrained cyclic 6-membered phosphate ring with the same number of charges as dA3 (Figure 4A). Consistent with the previously reported results, incubation of dA3 with Pep-KG gave heterogeneous fibrillar morphologies (Figure 4B), while TMP rapidly nucleates Pep-KG into the nanotube morphology seen with dsDNA and ssDNA templates (Figure 4C) (Rha et al., 2020).

These co-assemblies were also monitored for 12 h following dissolution *via* ThT fluorescence (Figure 4D). Here, the TMP-templated sample assembled more rapidly than dA3-templated and non-templated assemblies. Considering the distinct ordering of the charged phosphates in TMP, these data suggest that in addition to charged monomers, conformational charge ordering, in TMP covalently constrained, is most critical for nucleating paracrystalline ordering. Whereas dsDNA accelerates the formation of nanotubes relative to ssDNA but the DNA and Pep-KG assembly pathway is overall the same (Figure 3), the TMP and Pep-KG assembly pathway is totally distinct from the assemblies formed with dA3 (Figures 4B,C).

### Propagation of cross-*β* co-assemblies is sensitive to electrostatic interference

**FIGURE 5:**
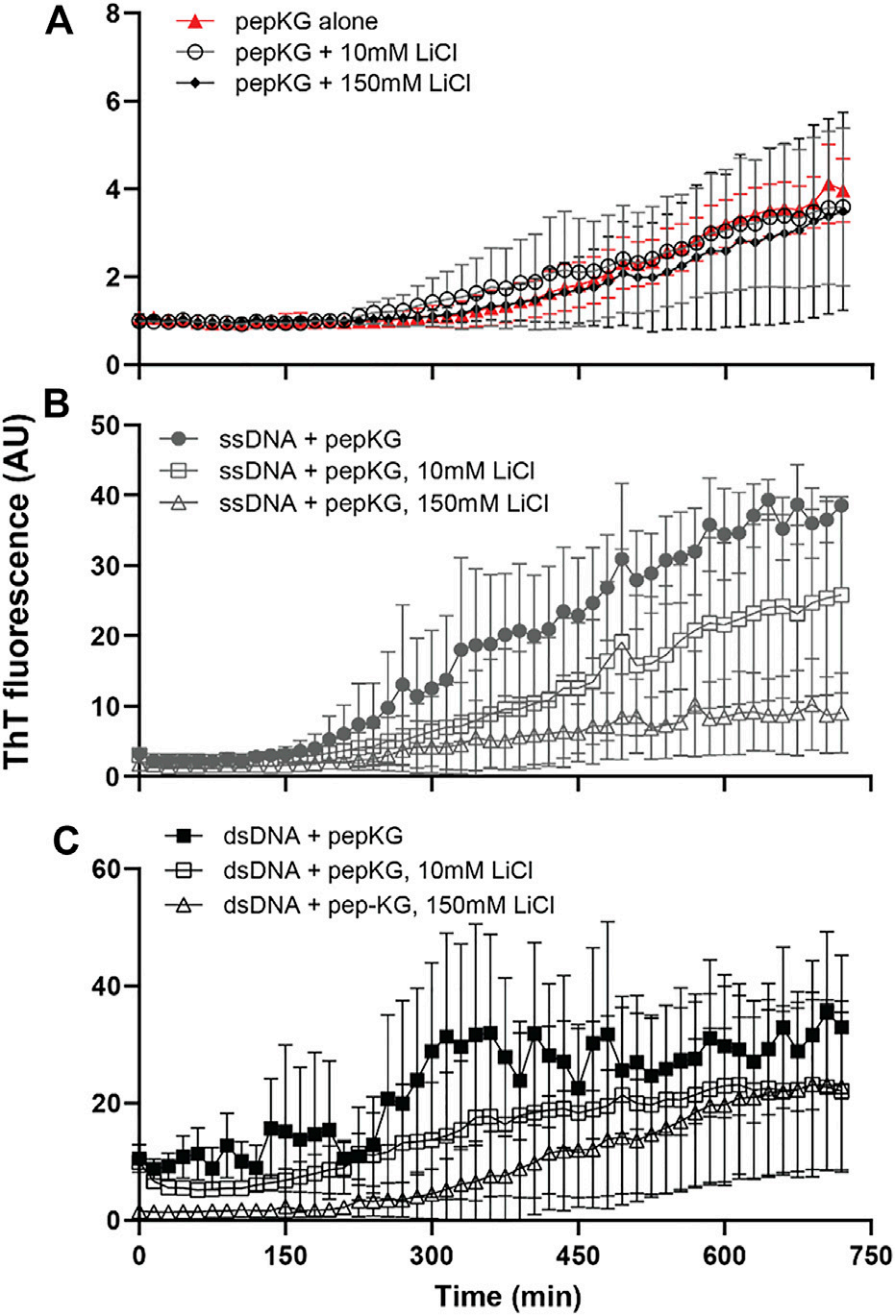
Pep-KG association with DNA can be modulated by LiCl. Cross-*β* assembly of Pep-KG, Pep-KG/ssDNA, and Pep-KG/dsDNA [**(A–C)**, respectively] monitored by ThT fluorescence in the presence of different LiCl concentrations, 10 and 150 mM. All assemblies were incubated in 40% MeCN at 37°C with dsDNA and ssDNA added at a 1:1 charge ratio, 41.6 μM dsDNA and 83.3 μM ssDNA, with 5 μM ThT that was added at the beginning of the assembly. It should be noted that, while ThT has been the standard probe for identifying the presence of cross-*β* assembly, it is unclear whether it plays a role in stabilization of the architecture. Thus, all assembly rates discussed here are relative to the baseline Pep-KG incubated with ThT. Error bars are 95% CI values and all samples are *n* = 3.

Given the electrostatic ordering achieved by dsDNA during templated peptide cross-*β* nucleation, we sought to compare ss/dsDNA as templates for propagation. Propagation rates have been obtained *via* imaging approaches, but because of the complexity these electrostatic-driven processes, we investigated propagation control with passivating salts. MgCl_2_ inhibits both nucleation and propagation in these co-assemblies, and here LiCl was selected because of Li^+^’s small size, single charge, and minimal impact on more complex DNA structures (Wen et al., 2014). Propagation was assessed *via* ThT kinetics at low and high concentrations of LiCl. The influence of LiCl concentrations on the relative growth rates showed little difference at high LiCl concentrations (Figure 5). This observation is most consistent with ssDNA and dsDNA being equally effective in co-assembly propagation in stark contrast to their effect on nucleation. Additional imaging approaches will be necessary to define these rates more precisely.

### Seeding with quadruplex DNA/peptide chimeras

**FIGURE 6:**
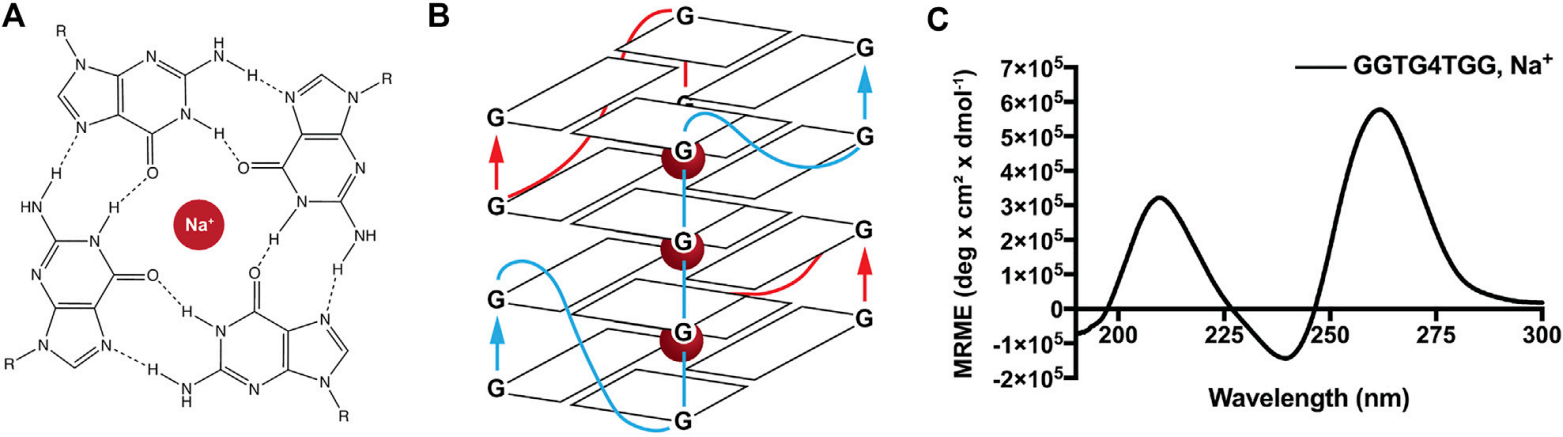
Ions like Na+ are conducive to guanine quadruplex formation. Metal cations, including K+ and Na+, facilitate guanine quartet formation through coordination with O6 atoms that project into the internal cavity **(A)**. Metal cations are arranged equidistant from guanine quartets in the quadruplex, aligning the eight oxygen atoms in a symmetric tetragonal bipyramidal configuration. The sequence GGTG4TGG used in this study requires two DNA strands to associate for proper quadruplex folding and gives the depicted parallel guanine quadruplex **(B)**. CD of GGTG4TGG in the presence of Na+ gives the expected parallel quadruplex signature with a maximum positive ellipticity at 260 nm **(C)**.

Given the effect of ordered charged templates on nanotube nucleation and the accommodation of ordered phosphate-containing molecules within propagating cross-*β* peptide assemblies, we sought to exploit these findings to construct functional sites along the peptide nanotube. Guanine quadruplexes (GQ) contain planes of four Hoogsteen base-paired guanine bases with phosphates defining the connecting edges. The bimolecular parallel quadruplex-forming DNA, GGTG4TGG, which requires two GQ DNA strands for assembly, organizes to form four stacked guanine quartets with well-ordered charges running along the corners of the GQ planes and potential for extended GQ stacking (Figures 6A,B). (Ilc et al., 2013; Do et al., 2017; Ahmed et al., 2018) As shown in Figure 6C, this DNA sequence assembles to give the characteristic CD signature of a parallel GQ. (Kypr et al., 2009; Bhattacharyya et al., 2016; Ahmed et al., 2018).

**SCHEME 1:**
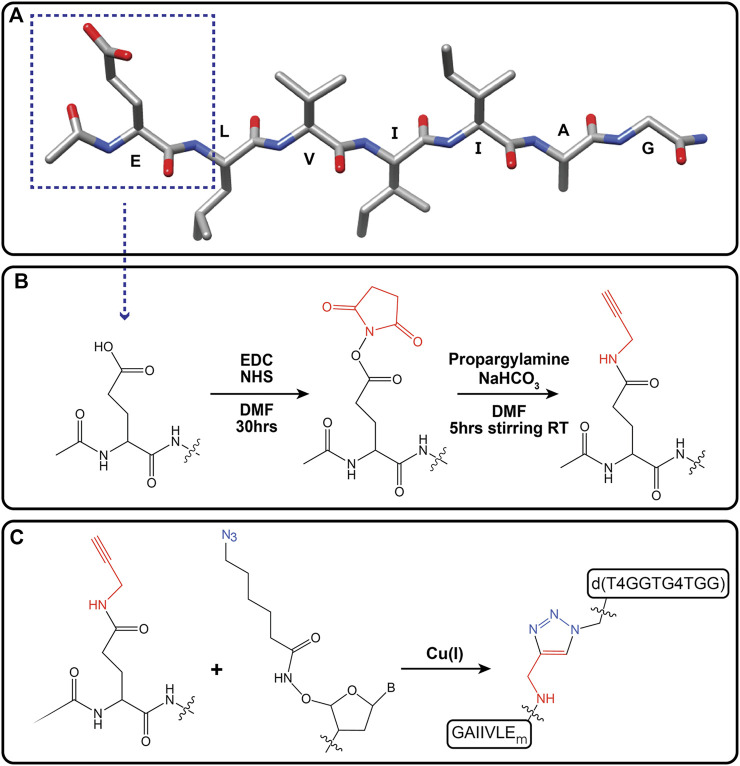
Alkynylation of terminal glutamic acid (Ac-EmLVIIAG-NH_2_) and click reaction with azide-modified guanine quadruplex DNA [d(T4GGTG4TGG)]. Primary peptide sequence for Ac-ELVIIAG-NH2. Dotted box outlines terminal glutamic acid residue **(A)**. Alkyne modification of carboxylic acid moiety of terminal glutamate *via* amide linkage (Ac-EmLVIIAG-NH2, where “m” denotes modification of glutamate) **(B)**. Cu(I) catalyzed click reaction of Ac-EmLVIIAG-NH2 and 5′-azide-modified ssDNA [d(T4GGTG4TGG)] gave the guanine quadruplex peptide conjugate (GQPC) through triazole formation **(C)**.

To enhance templating by this GQ template, GGTG4TGG was conjugated to the N-terminal residue side chain of the accompanying peptide *via* a Cu(I)-catalyzed click reaction. Initial peptide synthesis required replacement of the N-terminal lysine of Pep-KG with N-acetylated glutamic acid to give Ac-ELVIIAG-NH_2_. Further alkyne modification of the glutamic acid side chain was achieved following esterification using N-Hydroxysuccinimide (NHS) and amidation with propargylamine to give Ac-E_m_LVIIAG-NH_2_ (Scheme 1). Preparation of the GQ DNA included the addition of a 5′ azide-modified T4 linker, N_3_-TTTTGGTG4TGG. These alterations provided flexibility for chimera-associated quadruplex formation (T4 linker) and enabled peptide-DNA click-conjugation via triazole linkage (Scheme 1). (Hazel et al., 2004; Vondruskova et al., 2008; Do et al., 2017; Ahmed et al., 2018) The click reaction gave rise to the guanine quadruplex peptide conjugate (GQPC), with linkage between the modified glutamic acid sidechain and the 5′-end of the GQ DNA. To form the quadruplex, two strands of the GQPC chimera must hybridize to give Hoogsteen hydrogen bonds (Figure 6B), forming an intermolecular parallel guanine quadruplex with four quartets and thymine nucleotides present in the loops.

**FIGURE 7:**
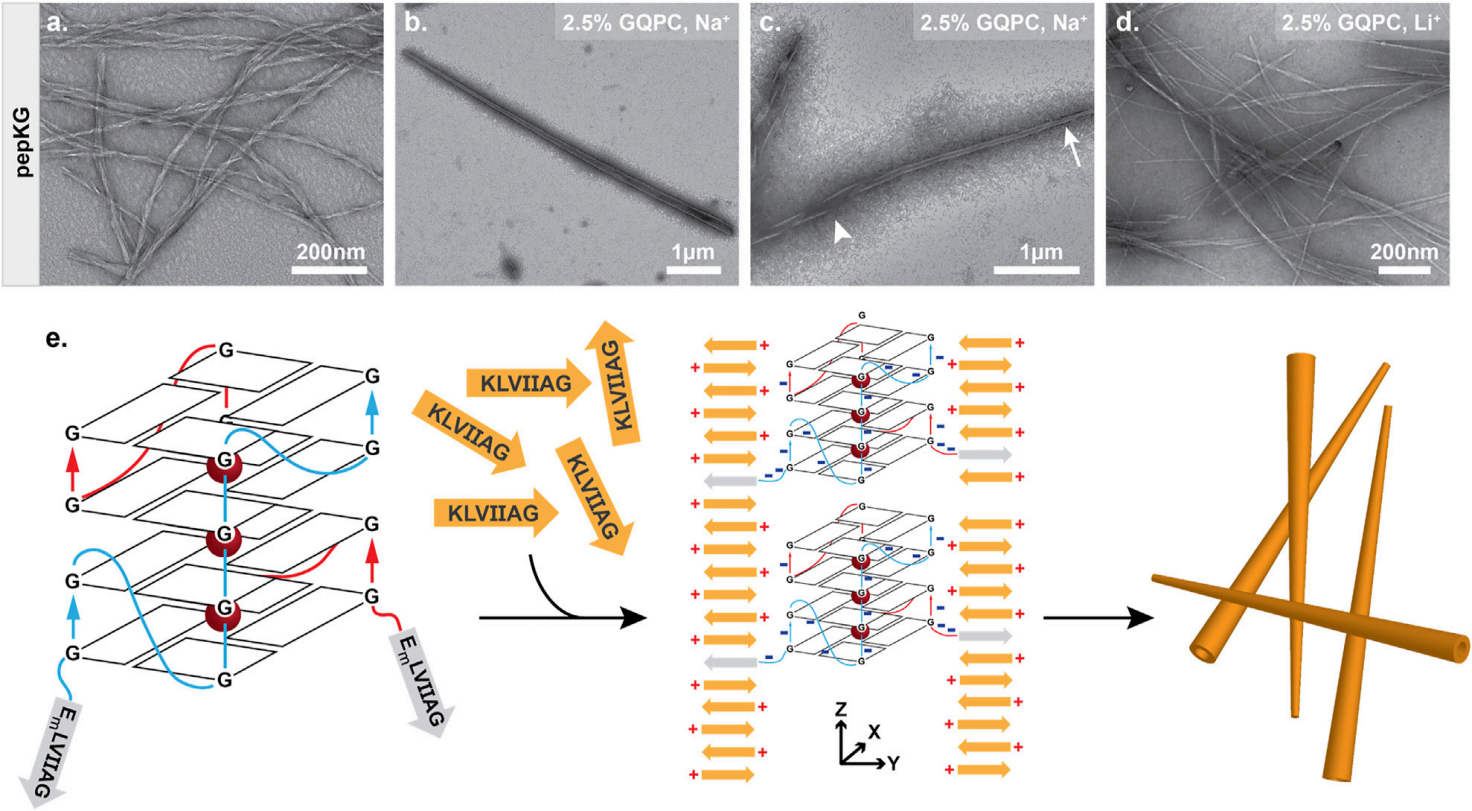
Guanine quadruplex folding is necessary for tapered nanotube morphology. Pep-KG (0.4 mM) assembled in sodium phosphate buffer results in bundled fiber formation **(A)**. Co-assembly of GQPC (2.5% molar equivalent) and pep-KG (0.4 mM) yields tapered conical assemblies resembling nanotubes in sodium phosphate buffer **(B)**. Like previously described nanotubes, GQPC/Pep-KG co-assemblies form ribbons first. The tapered ends of GQPC/pep-KG co-assemblies close first (tapered end, arrow; wide end, arrowhead) **(C)**. LiPO_4_ buffer used in place of NaPO_4_ resulted in a mixture of both non-tapered nanotubes and fibers **(D)**, suggesting that GQ formation is essential for conical tube morphology as depicted in the proposed co-assembly mechanism **(E)**.

To improve the propensity for supramolecular assembly and direct quadruplex formation, peptide concentrations and temperature were optimized. We found that below 0.4 mM, Pep-KG nucleates slowly and when combined with GQPC, produced few visible assemblies by electron microscopy. At concentrations above 0.4 mM, pep-KG and GQPC gave heterogenous co-assemblies. Therefore, 0.4 mM Pep-KG was used during the co-assembly process (Figure 7A). Due to the small reaction sizes, a maximum concentration of 10 μM GQPC was achieved and combined completely with 0.4 mM pep-KG. Pep-KG and GQPC were combined in sodium phosphate buffer (pH 7.4), heated to 95°C for five minutes to melt any existing nuclei, and returned to room temperature by decreasing the sample temperature at a rate of 2°C/minute to promote quadruplex formation. GQPC/pep-KG co-assemblies, in which the charge ratio was not 1:1 but rather the overall concentration ratio was 40:1, resulted in conical nanostructures by TEM (Figures 7B,C). Similar to dsDNA and ssDNA-directed assembly, GQPC-templated assembly initially formed ribbons with the narrow end of the conical nanotubes closing first (Figure 7C).

The resulting conical nanotube is distinct from the multilamellar nanotubes found with ssDNA, dsDNA, or TMP templates. Most notably, the chimeric template nucleated GQPC/Pep-KG co-assembly at low concentrations (10 μM GQPC) and formed nanotubes with a width greater than that observed for either the ssDNA or dsDNA template visualized by TEM. Cross-*β* nanotube assemblies arise from growth along the lamination (sheet stacking) axis as well as the *β*-sheet H-bonding axis, and the covalently attached GQ-DNA was designed to impact nucleation along both growth axes by favoring initial GQ assembly.(Lu et al., 2003; Mehta et al., 2008) We hypothesize that early selection for guanine quadruplex formation coupled with low GQPC concentration, positioned GQPC conjugates at the tapered end of mature conical assemblies and facilitated further GQPC/Pep-KG templating. Future studies will be required to further elucidate the structural characteristics of the GQPC/Pep-KG co-assemblies. In addition to favored GQ formation, the sodium phosphate buffer may promote nanotube assembly through neutralization of the leaflet interface, similar to the previous study by Li et al. where salt passivation of nanotube bilayers was documented (Li et al., 2016). Electrostatic force microscopy (EFM) of GQPC/Pep-KG co-assemblies was consistent with a strong positive external surface charge for the tapered nanotubes, placing the positive N-terminal lysine of Pep-KG on the surface and sequestration of the quadruplex DNA within the nanotube interior (Supplementary Figure S9).

**FIGURE 8:**
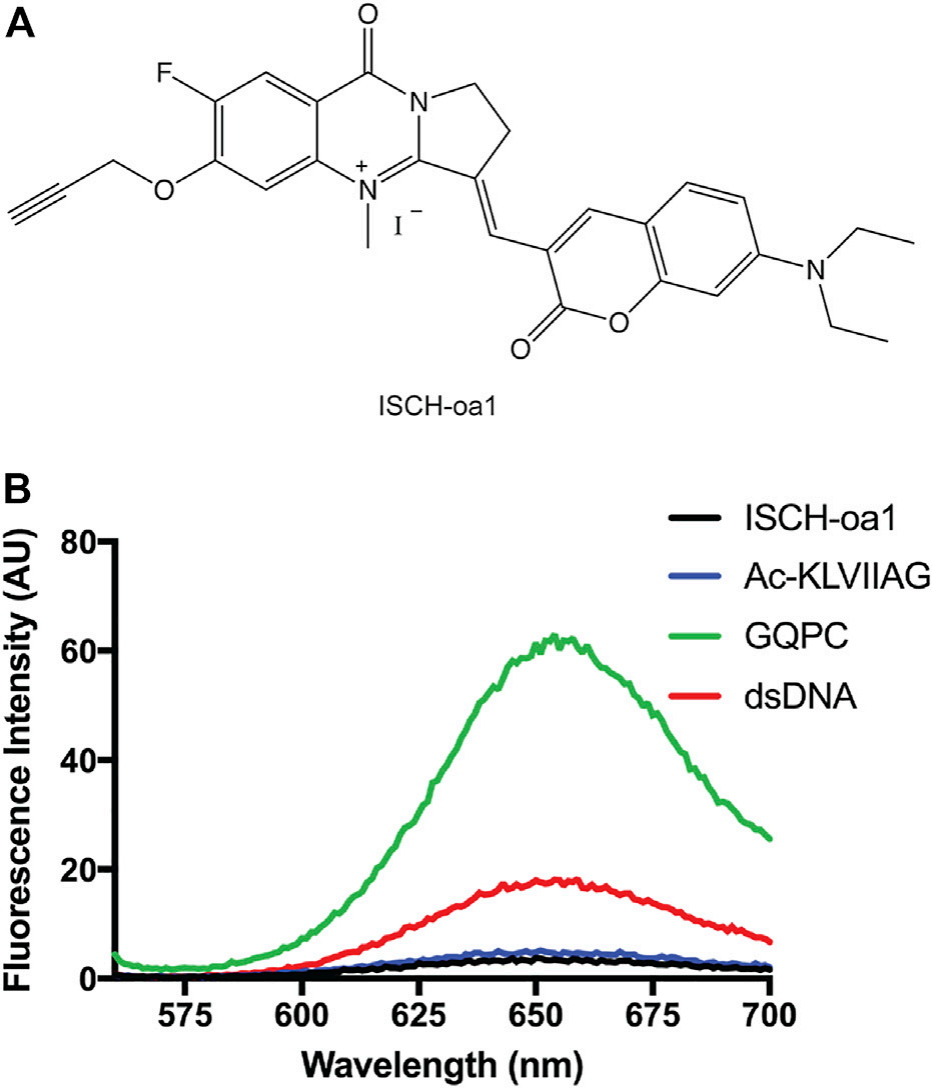
Guanine quadruplexes are present in GQPC/Pep-KG tapered nanotubes. ISCH-oa1 **(A)** was used as a fluorescent marker of guanine quadruplex folding and presence in conical co-assemblies. In the presence of GQ, ISCH-oa1 showed a marked fluorescence increase relative to ISCH-oa1 alone or in the presence of other DNA moieties **(B)**. GQPC indicates GQPC/pep-KG co-assemblies formed in the presence of Na+. Ac-KLVIIAG indicates pep-KG.

GQPC/Pep-KG nanotubes contain strong peptide *β*-sheet CD signatures (Supplementary Figure S4), however the GQ seeds are below the limit of detection, thus fluorescent spectroscopy was used to probe GQ formation. As shown in Figure 8, when combined with GQPC/Pep-KG co-assemblies the quadruplex-specific fluorophore ISCH-oa1 gives a 16-fold increase in fluorescence over Pep-KG assemblies or dye alone and a 4-fold increase in fluorescence over a dsDNA control (Chen et al., 2016). Unlike sodium, lithium does not stabilize quadruplex assembly, and co-assembly of GQPC/Pep-KG in lithium phosphate buffer results in heterogeneous, thin-walled nanotubes and fibers without conical tube tapering (Figure 7D) (Venczel and Sen, 1993; Bhattacharyya et al., 2016). Altogether, these results support the incorporation of GQPC and assembly of parallel GQs within the conical GQPC/Pep-KG co-assemblies (Figure 7E).

## Conclusion

Varying ATP concentrations in cells can work to control protein condensation, (Patel et al., 2017; Aida et al., 2022; Saurabh et al., 2022) and biphasic control of PLD proteins like FUS in membraneless compartments relies on electrostatic and π-cation interactions that, in a concentration-dependent manner, both condenses and dissolves protein condensates (Patel et al., 2017; Ren et al., 2022). Phosphate salts are also strongly kosmotropic and are known to induce crystalline order in other biomolecular condensates (Malay et al., 2020). Furthermore, previous work has used the nucleating core of the Aβ peptide of Alzheimer’s disease as a scaffold to elucidate the importance of electrostatic interactions in nucleic acid/amyloid co-assembly (Rha et al., 2020). This motif forms an initial biomolecular condensate which biases conformational sampling, enabling both self-templating and pre-organizing the condensate for external templated assembly (Chen et al., 2017; Rengifo et al., 2020). Most interestingly, while ssDNA and RNA effectively templated precise peptide ordering, the nucleic acid had no higher-order structure and this motivated our investigation of more ordered templates. Based on this evidence, a minimal model was developed to explore the importance of polyanion structural ordering in the formation and selection of nuclei during liquid to solid transitions.

Our model system now establishes that structured nucleic acid assemblies provide a significantly lower threshold for effective templating as well as provide a framework for exploring the structural rules underpinning the early dynamics of membraneless organelles. Specifically, we find that the different ordering of phosphate groups on a polyanionic template modulates the liquid to solid phase transition from coacervate droplets to paracrystalline assemblies, either accelerating the formation of a stable nucleus which propagates out of the condensate or totally altering which nuclei is selected for within the condensate. This finding highlights the versatility of the cross-*β* architecture in accommodating various single-stranded and higher-order nucleic acid structures. Furthermore, our experiments with non-stoichiometric amounts of peptide-DNA chimera allow for seeding of distinct structures beyond co-assemblies, effectively separating nucleation with environmental agents from propagation. Finally, in the rapidly evolving field of biomaterials, the utilization of a specific nucleating material for nucleation and a different monomer for paracrystalline assembly propagation may provide avenues for design and incorporation of unique functional sites along amyloid-mimetic materials.

## Data Availability

The raw data supporting the conclusion of this article will be made available by the authors, without undue reservation.
