# Supplementary material for: Polyanion order controls liquid-to-solid phase transition in peptide/nucleic acid co-assembly
Source: Front Mol Biosci. 2022 Nov 14;9:991728. doi: 10.3389/fmolb.2022.991728 (PMC9702359; doi:10.3389/fmolb.2022.991728)
Supplement: Supplementary file 1 [file DataSheet1.PDF]

Supplementary Material

1 Supplementary Data

HPLC purification of pep-KG and pep-EG

Purification of both Pep-KG and Pep-EG was carried out using a Waters 2545 Quaternary Gradient Module (Serial # HO845Q016R) and a Waters 2998 Photodiode Array Detector (Serial # M12998467A) on a C18 reverse-phase HPLC column (XSelect CHS Prep. C18 5µm OBD 19x250 mm Column PN:186005492 SN:162|3801711405). Pep-KG was purified with a gradient from 25% to 35% acidified acetonitrile (MeCN , 0.1% TFA) in acidified HPLC water (with 0.1% TFA). Pep-KG eluted reliably at 31% MeCN (**Figure S6**) as verified by ESI (**Figure S7**). Pep-EG was purified with a 28%-33% gradient of MeCN in 25 mM TEAA (pH 7) HPLC water. Pep-EG eluted as two peaks at 28.6% and 28.7-28.8% MeCN, but only the second peak was considered pure, as verified by ESI (**Figure S9**) and collected (**Figure S8**). HPLC purification of Ac-E_mLVIIAG-NH_2 was attempted but yielded poor separation, shown by ESI (**Figure S5**). Purified peptides were rotary evaporated and then freeze dried for preparation of the desalting process.

Desalting of Pep-KG

Pep-KG and Pep-EG were each desalted with Sep-Pak C18 3mL cartridges (Waters Co., Milford, M) by first dissolving the purified peptide in HPLC water with 0.1% TFA added, using 6 mL of acidified water per 0.1 mmol peptide and sonicating. Before adding dissolved peptide, each Sep-Pak cartridge was prepared by adding 3 mL of MeCN + 0.1% TFA twice, followed by 2 additions of 3 mL of water + 0.1% TFA. After this preparatory step, 3 mL of dissolved peptide was added to each cartridge and the filtrate from this peptide was collected and passed through the cartridge again. Next, 3 mL of water with 0.1% TFA was added to the cartridges to remove salts from the peptide. After washing, peptides were eluted off the column with two additions of 3 mL 50% MeCN solution with 0.1% TFA. MeCN was removed from the eluted peptide fraction via rotary evaporation, where the aqueous solution was then frozen for lyophilization.

DNA sequences

All DNA was purchased from Integrated DNA Technologies with 5' phosphorylation (/5Phos/) modification and standard desalting for purification. The Drew-Dickerson dodecamer sequence was as follows: 5'-CGCGAATTCGCG-3'. dsDNA Drew-Dickerson dodecamer was ordered as a duplex, while ssDNA Drew-Dickerson dodecamer was ordered as single strands. PolyA3 was custom-ordered due to the company's ordering restriction for oligomers fewer than 5 nucleotides.

2 Supplementary Figures and Tables

For more information on Supplementary Material and for details on the different file types accepted, please see here. Figures, tables, and images will be published under a Creative Commons CC-BY licence and permission must be obtained for use of copyrighted material from other sources

(including re-published/adapted/modified/partial figures and images from the internet). It is the responsibility of the authors to acquire the licenses, to follow any citation instructions requested by third-party rights holders, and cover any supplementary charges.

2.1 Supplementary Figures

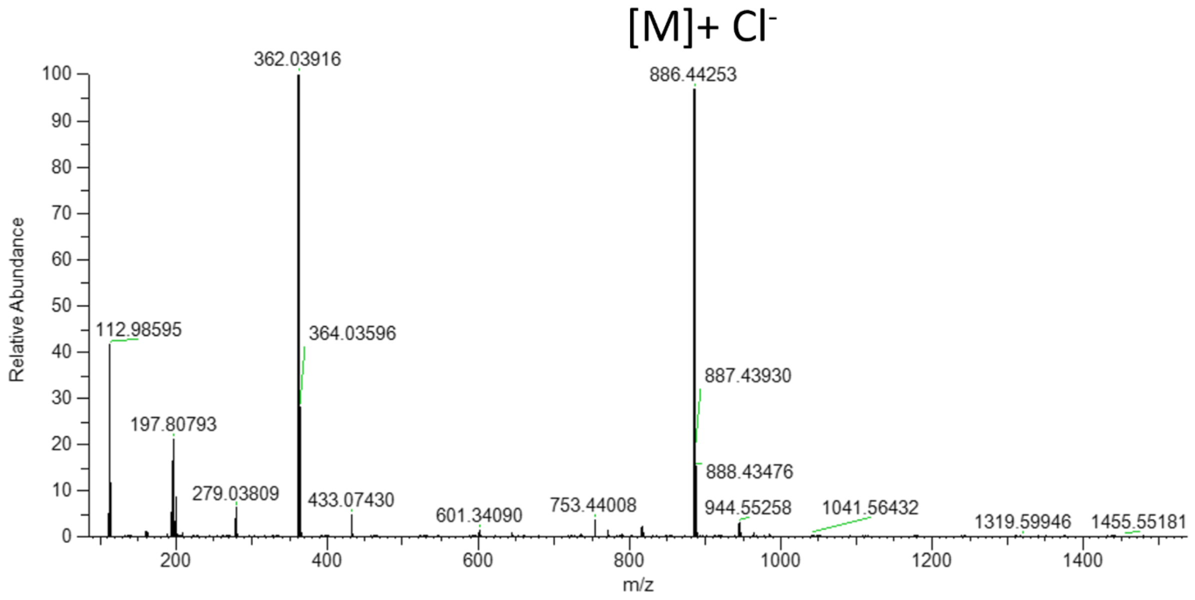

Figure S1. ESI spectra of peptide imide product generated by EDC/NHS coupling with Pep-EG

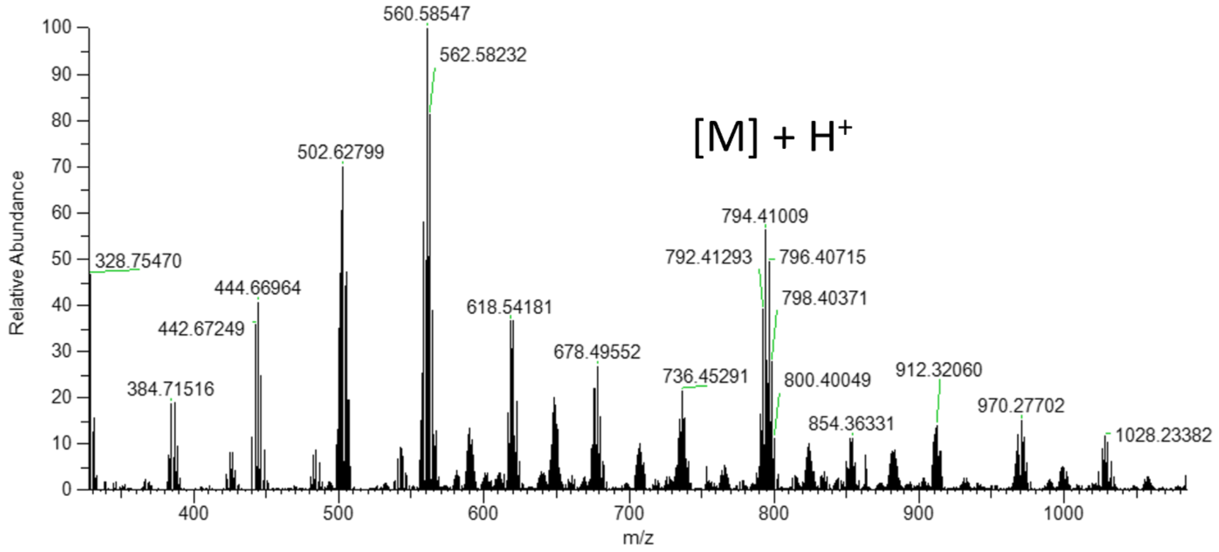

Figure S2. ESI spectra of alkynylated pep-EG after reacting crude imide peptide with propargylamine

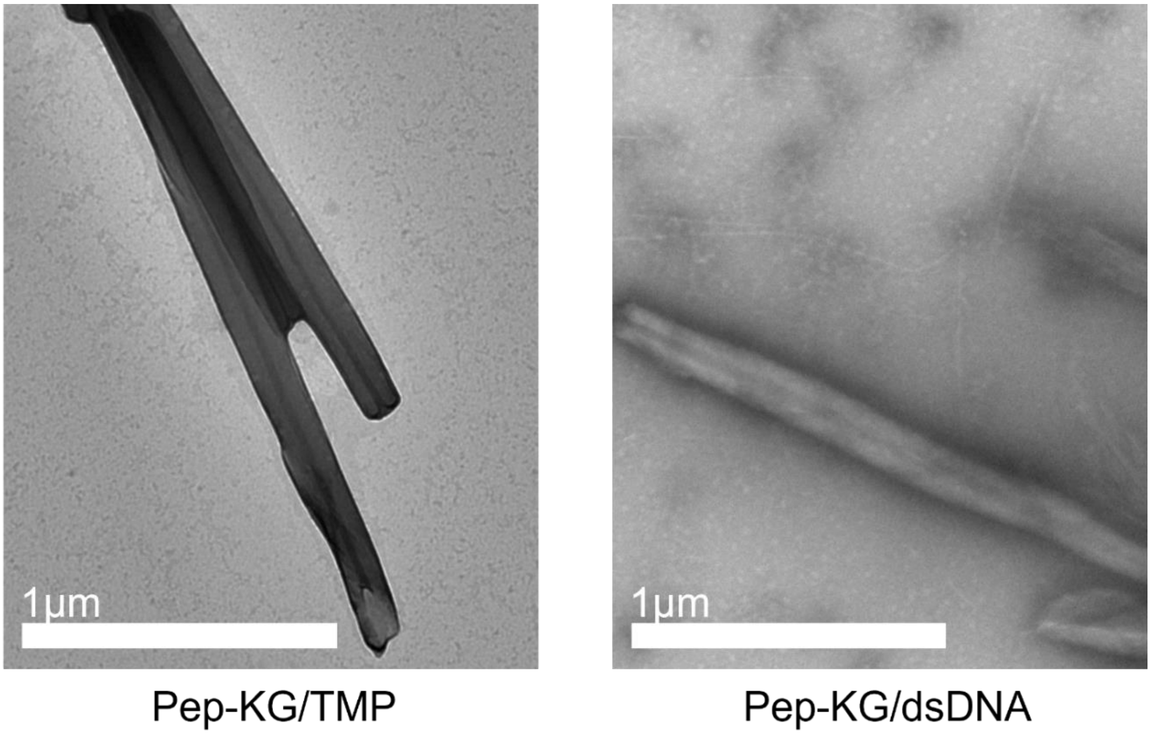

Figure S3. (left) Co-assembly of pep-KG (1 mM) with sodium trimetaphosphate (TMP, 333 μM). (right) Co-assembly of pep-KG with dsDNA (41.7 μM). Both figures show thick-walled nanotube morphology.

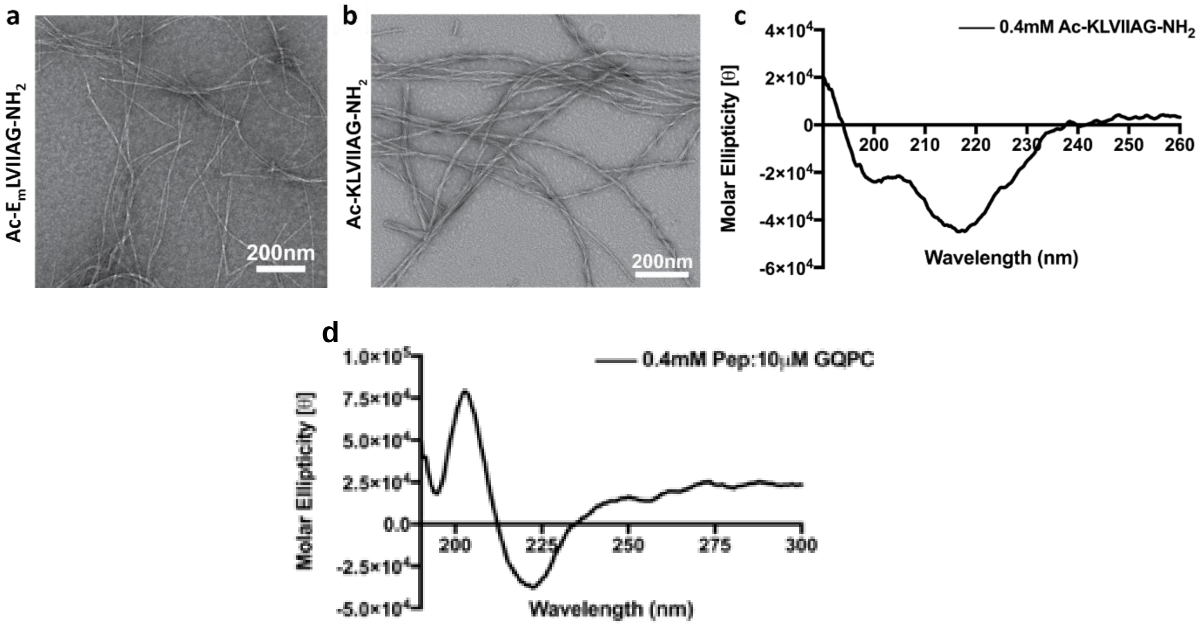

Figure S4. TEM images of assembled Ac-E_mLVIIAG-NH_2 (a) and pep-KG (b) showing fiber morphology. c. CD spectra of pep-KG at slightly above its critical peptide assembly concentration of 0.3 mM. d. CD spectra of GQPC/pep-KG, a pep-KG assembly seeded with 2.5% GQPC relative to overall pep-KG concentration.

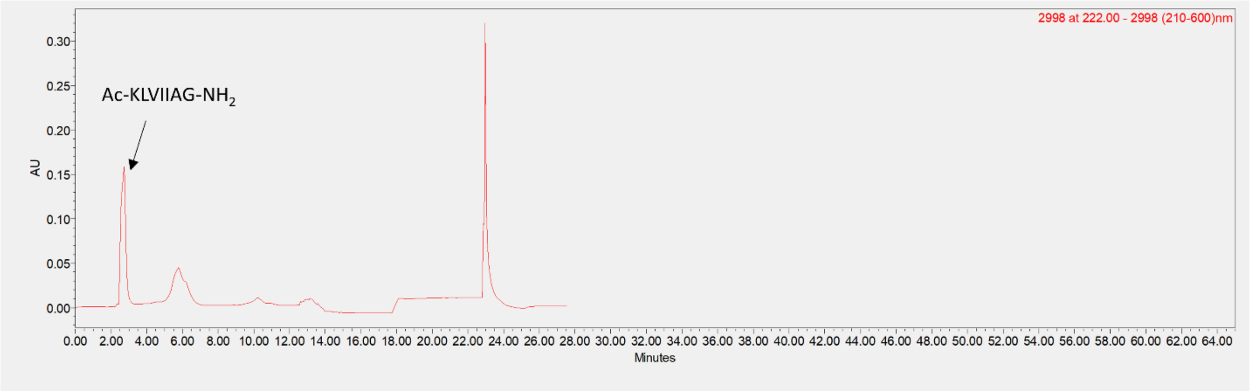

Figure S5. HPLC purification of Ac-KLVIIAG-NH_2, where the peptide elutes at 31% acidic MeCN

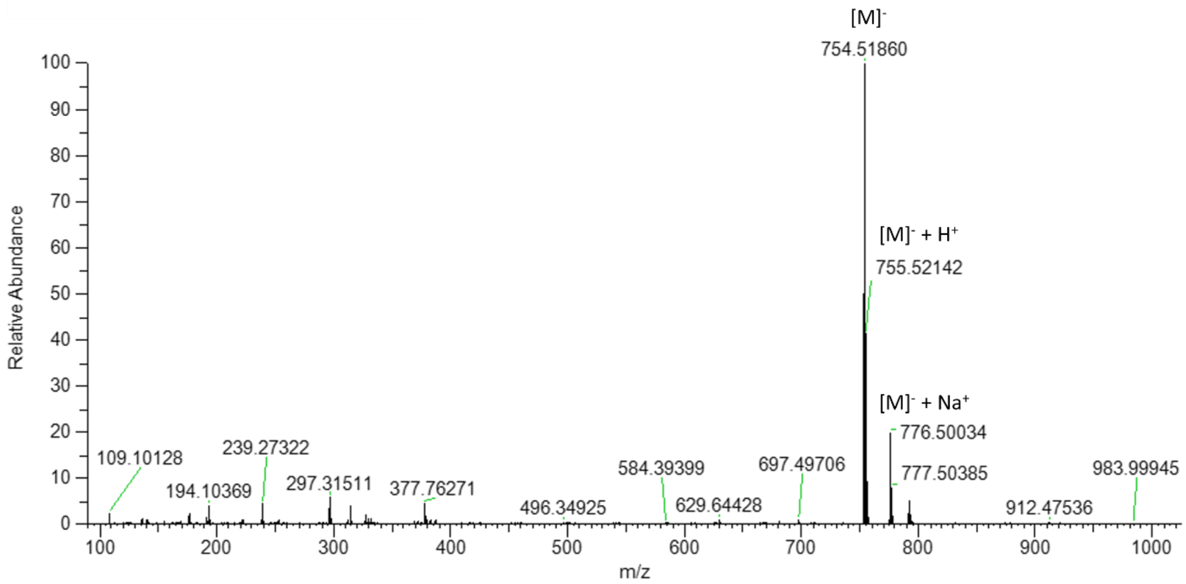

Figure S6. ESI spectra of Ac-KLVIIAG-NH_2 after HPLC purification

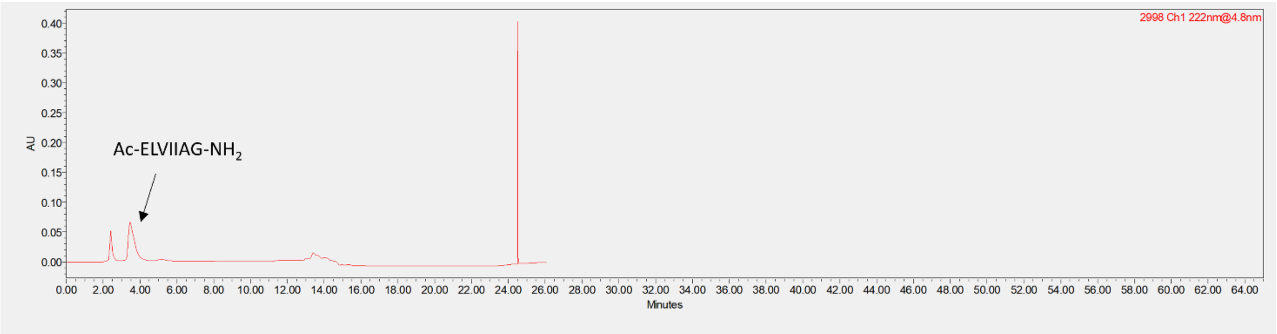

Figure S7. HPLC purification of Ac-ELVIIAG-NH_2, where the peptide elutes at 29% MeCN

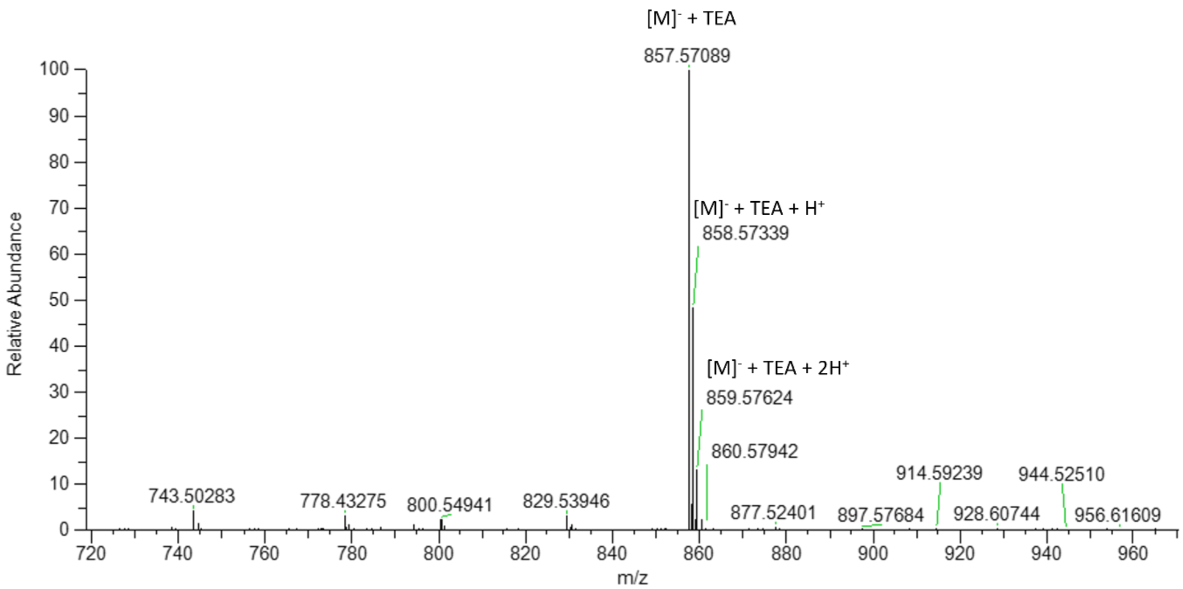

Figure S8. ESI spectra of Ac-ELVIIAG-NH_2 after HPLC purification

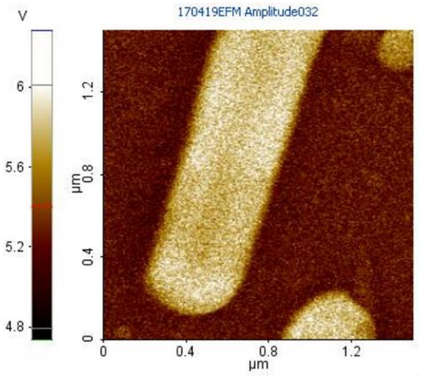

Figure S9. EFM of mature GQPC/Pep-KG co-assemblies.

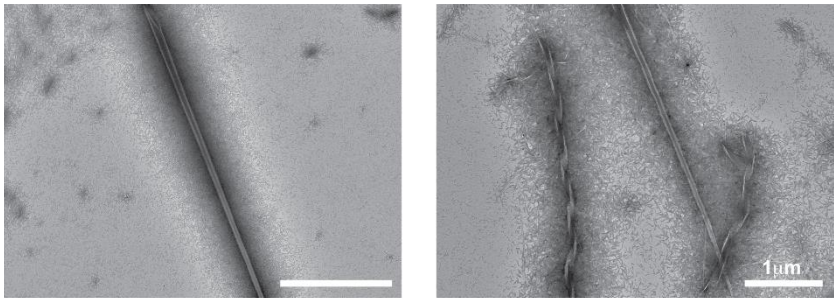

Figure S10. TEM images of GQPC/pep-KG assembled in 40% MeCN at 37°C after 7 day incubation.
